# Menstrual hygiene practice among female adolescents and its association with knowledge in Ethiopia: A systematic review and meta-analysis

**DOI:** 10.1371/journal.pone.0254092

**Published:** 2021-08-04

**Authors:** Melaku Yalew, Bezawit Adane, Mastewal Arefaynie, Bereket Kefale, Yitayish Damtie, Kefale Mitiku, Amare Agmas, Gebeyaw Biset, Tilahun Dessie Alene, Metadel Adane, Elsabeth Addisu, Reta Dewau

**Affiliations:** 1 Department of Reproductive and Family Health, School of Public Health, College of Medicine and Health Sciences, Wollo University, Dessie, Ethiopia; 2 Department of Epidemiology and Biostatistics, School of Public Health, College of Medicine and Health Sciences, Wollo University, Dessie, Ethiopia; 3 Department of Physiology, School of Medicine, College of Medicine and Health Sciences, Wollo University, Dessie, Ethiopia; 4 Department of Anesthesia, School of Medicine, College of Medicine and Health Sciences, Wollo University, Dessie, Ethiopia; 5 Department of Pediatrics Nurse, School of Nursing and Midwifery, College of Medicine and Health Sciences, Wollo University, Dessie, Ethiopia; 6 Department of Pediatrics, School of Medicine, College of Medicine and Health Sciences, Wollo University, Dessie, Ethiopia; 7 Department of Environmental Health, College of Medicine and Health Sciences, Wollo University, Dessie, Ethiopia; MAMTA Health Institute for Mother and Child, INDIA

## Abstract

**Background:**

Previous studies on menstrual hygiene practice were largely inconsistent and single national evidence is required. Thus, this study aimed to assess the pooled prevalence of menstrual hygiene and its association with knowledge about menstrual hygiene among female adolescents in Ethiopia.

**Methods:**

The study was designed based on the Preferred Reporting Items for Systematic Reviews and Meta-Analysis Protocols (PRISMA-2015 Guidelines). This systematic review included studies conducted on female adolescents in Ethiopia irrespective of their publication and study period until the end of July 1, 2020. The data extracted in the Microsoft Excel sheet format was exported into the STATA/SE14 version statistical software for further analysis. I^2^ test was used to test heterogeneity and publication bias was assessed by using Egger’s weighted regression test.

**Results:**

Thirteen full-text articles including 6907 participants were included in this systematic review and meta-analysis. Using the random effect model, the pooled prevalence of poor menstrual hygiene practice was 48.98% [95% CI: (36.42, 61.53)]. Those female adolescents who had poor knowledge were 2.6 times more likely to have poor menstrual hygiene practice as compared to counterparts [AOR = 2.61, 95% CI: (1.45, 4.72)].

**Conclusions:**

The prevalence of poor menstrual hygiene practice was high and knowledge regarding menstrual hygiene was significantly associated with poor menstrual hygiene practice. Information education communication and behavioral change communication at all levels of education should be the primary focus area of the government.

## Introduction

World Health Organization (WHO) defines adolescence as a time of transition from childhood to adulthood. In terms of demographic definition, they are a group of people age ranged from 10 to 19 years [1]. Globally, 52% of females (26% of the total population) were covered by reproductive age. Adolescent girls also constitute one-fifth of the total female population of the world [2, 3]. Menstruation is still surrounded by social taboos, supernatural beliefs and misconceptions even if it is a normal and physiological process of women [4, 5]. Due to this, menstrual hygiene practice is a common problem of adolescent girls mainly in developing countries [6–8]. Poor menstrual hygiene practice has a pronounced effect on the quality of health, education and other aspects of puberty [6, 7, 9–11]. It may complicate and lead to reproductive tract infection unless it is properly managed [11–14]. Mensuration especially poor hygiene practice attributed to 40% of absenteeism in school [11, 15, 16]. Poor menstrual practice is also connected with stillbirth, miscarriage, infertility and cervical cancer [17].

The prevalence of poor menstrual hygiene practice was 27.5 to 40% in Nepal studies [18, 19] and 68.5% in Bangladesh [20]. It was also ranged between 44.8–81.7% in different studies conducted in India [21–24]. The prevalence of poor menstrual hygiene practice was 45.45% in Uganda [25], 74.7% in Nigeria [26], 28.8% in Kenya [27] and 69.9% in Ghana [28]. It was estimated to be 9% to 76% in Ethiopia studies [29, 30].

Numerous studies showed that very a high number of girls start menstruating without having any idea of what is happening to them and why it is happening [31–33]. Menstrual hygiene practice could be influenced by female characteristics (age, age at menarche, residence, knowledge) [23, 25, 27, 34–38], parental factors (education, occupation, open communication, income) [23, 25, 27, 34, 35, 37, 38] and access to water [12, 39, 40].

Even if menstrual hygiene practice was previously studied in Ethiopia, those studies were inconsistent and there was no single conclusive finding regarding the prevalence and effect of knowledge on menstrual hygiene practice in Ethiopia. In addition, prior systematic review and meta-analysis has not been conducted and public health experts and policymakers who are working with adolescents need updated evidence regarding menstrual practice. Therefore, this study aimed to assess the pooled prevalence of menstrual hygiene practice and its association with knowledge among female adolescents in Ethiopia.

## Methods

### Study design and search strategy

The study was designed based on the Preferred Reporting Items for Systematic Reviews and Meta-Analysis Protocols (PRISMA-Guidelines) [41] (see S1 File). The following databases were searched systematically: Medline/PubMed, CINAHL, Cochrane Central Library, HINARI, Global Health and Google scholar from May 12 to July 1, 2020. The articles were searched using key terms developed according to Medical Subject Heading (MeSH): (“Menstruation”, “menstrual hygiene”, menstrual hygiene practice”, knowledge on menstrual hygiene practice”, “factors”, “factors associated”, “risk factors”, “predictors”, “females”, “school girls”, “women” and “adolescents”. All the key terms were used individually and in combination through Boolean operators (“AND”/ “OR”) as necessary (see S2 File). The search was done by two authors (MY and BA independent).

### Study selection

**Inclusion criteria**
- Setting: Studies conducted at either facility or community level.
- Outcome: Studies conducted menstrual hygiene practice as a primary outcome.
- Publication: Either published in peer-reviewed journals or unpublished studies.
- Time frame: all studies irrespective of data collection and publication year until the end of July 1, 2020
- Language: studies published only in English language were included in this review.

**Exclusion criteria**
- Studies in which the outcome was not clearly reported
- Studies which were pure qualitative were excluded from systematic review and meta-analysis.

### Variable measurement

The outcome variable (poor menstrual hygiene practice) was measured using ten items “YES” or “NO” questions. The response of each item was measure “1” for correct answers ad “0” for I don’t know or wrong answers. Individuals with a total sum score of less than 50% were classified as had poor menstrual hygiene practice, otherwise good practice [19, 20, 30, 42–44]. Similarly, knowledge was measured using eight items. The response of each item was measure “1” for correct answers ad “0” for I don’t know or wrong answers. Individuals with a total sum score of less than 50% were classified as had poor knowledge, otherwise good knowledge [30, 43, 45–47].

### Quality assessment and data extraction

Those articles identified in all databases were exported to Endnote X8 and duplicate files were excluded. The remaining articles and abstracts were independently screened by two groups (MA and BK) for inclusion in the full-text appraisal. It was assessed using Joanna Brigg’s Institute (JBI) critical appraisal checklist according to the study design of each article [48, 49]. Two independent authors (YD and KM) assessed the quality of the articles and the differences in the scales result were settled by taking the average result of both reviewers (see S3 File). Data were extracted using Microsoft excel 2010 sheet and the sheet contained the following list of variables: authors name followed by initials, year of study, year of publication, study setting, study design, sample size, response rate, quality score, region, poor menstrual hygiene and knowledge as a factor (extracted in the form of two by two tables sequentially labeled as A, B, C, D representing the four cells of two by two table) to calculate odds ratio. Two authors (EA and RD) extract the data and any disagreements between the two reviewers during extractions were solved through discussion and consensus.

### Data synthesis and statistical analysis

The data extracted in the Microsoft Excel sheet format was exported into the STATA/SE14 version statistical software for analysis. The pooled effect of the point estimate of poor menstrual hygiene practice in Ethiopia was calculated by DerSimonian & Liard’s method of random effect model at P-value less than 0.05 [50]. Statistical significant heterogeneity with I^2^ tests greater than 75% was taken as high heterogeneity and it was subjected to sub-group and sensitivity analysis. Finally, publication bias was assessed by using Egger’s weighted regression test method with a p-value < 0.05 was considered as significant publication bias [51].

## Results

### Study selection

**Fig 1:**
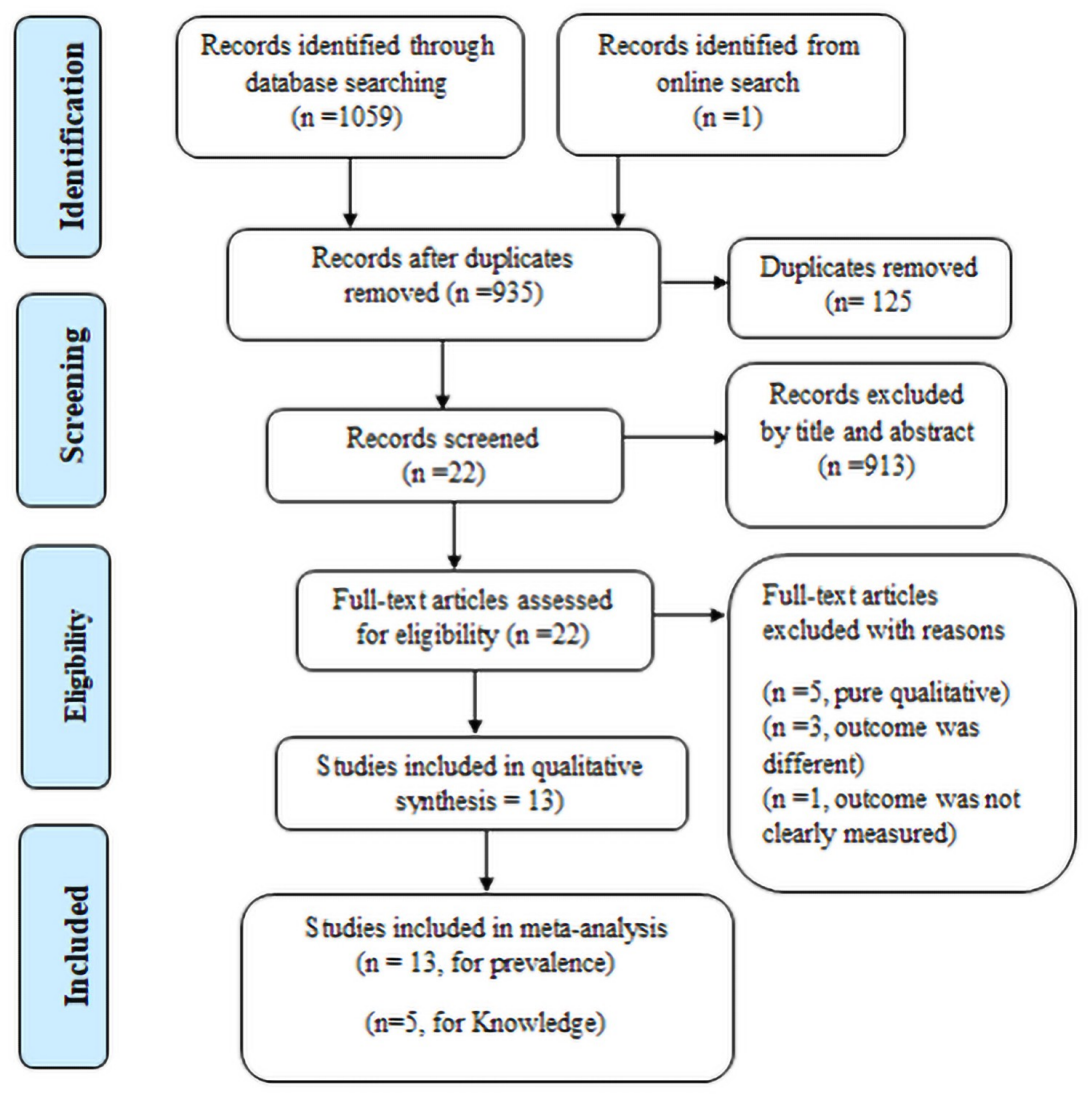
PRISMA flow diagram of menstrual hygiene practice and its association with knowledge, a systematic review and meta-analysis, Ethiopia, 2020.

A total of 1060 articles were identified from all databases (PubMed, Cochrane Library, CINAHL, HINARI, Global Health and Google scholar). Of which, 125 were excluded due to duplication, 913 through review of titles and abstracts. In addition, 9 full-text articles were excluded due to different reasons. Finally, 13 full-text articles were found to be eligible in systematic review and meta-analysis (Fig 1).

### Characteristics of included studies

The total number of participants included in this systematic review and meta-analysis were 6907 which was varied from 274 to 1006 in a study conducted in Oromia [52] and Amhara regional state respectively [30]. Five of the included articles were conducted in Oromia region [44, 52–55], four from Amhara [29, 30, 45, 46], one from Addis Ababa [43], one from Harari [47], one from Tigray [56] and one from Southern Nation Nationality and Peoples [42]. All the studies included in the review were cross-sectional study design (Table 1).

**Table 1:** Summary characteristics of studies included in systematic review & meta-analysis.

Authors	Study year	Year of Publication	Regions	Study setting	Sample Size	Prevalence of poor practice	RR%	Quality score
Belayneh Z et al	2018	2019	SNNP	High	791	60.30	98.10	8
Felleke A et al	2019	.	Harari	High	301	44.18	100.00	5
Fisseha M et al	2014	2017	Amhara	High	423	69.97	100.00	6
Gedefaw G et al	2019	.	Amhara	High	409	51.10	96.70	7
Gultie T et al	2013	2014	Amhara	High	492	9.10	100.00	6
Upashe S et al	2014	2015	Oromia	High	828	60.14	98.00	8
Biruk E et al	2017	.	Addis Ababa	Both	756	47.49	98.00	7
Bekele F et al	2017	2018	Oromia	High	274	33.21	100.00	6
Anchebi T et al	2016	2017	Oromia	High	398	42.96	94.30	7
Shallo S et al	2018	.	Oromia	High	336	53.57	92.30	5
Kitesa B et al	2016	2016	Oromia	High	430	29.77	100.00	6
Azage M et al	2015	2018	Amhara	Community	1006	75.55	99.60	8
Berhe H et al	2013	2018	Tigray	High	463	59.18	97.00	6

Both-Primary and high school, High- High school, RR-Response rate and SNNP- Southern Nation nationalities and peoples.

### Prevalence of menstrual hygiene practice in Ethiopia

**Fig 2:**
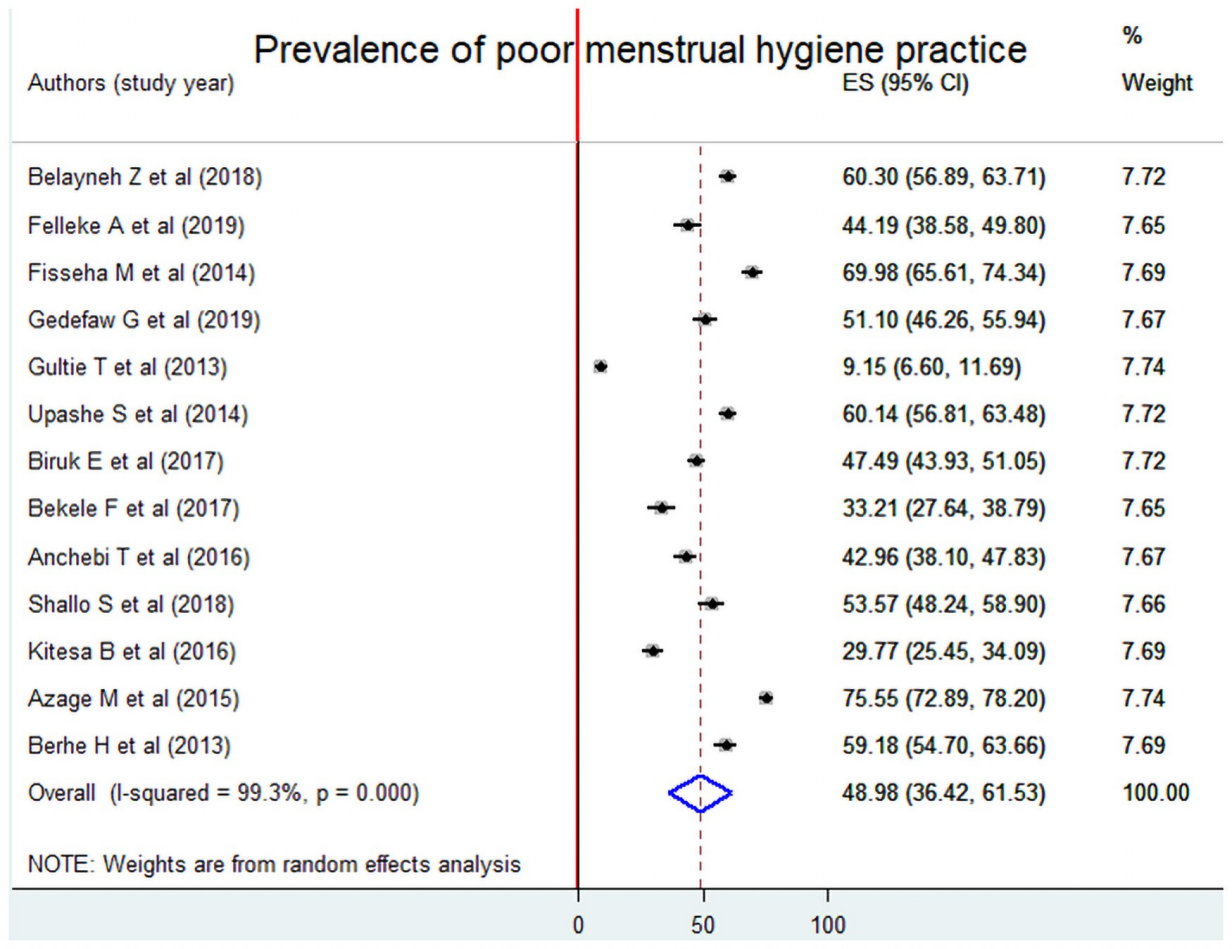
Forest plot of the pooled prevalence of poor menstrual hygiene practice using the random effect model, a systematic review and meta-analysis, Ethiopia, 2020.

The pooled prevalence of poor menstrual hygiene practice was 48.98% [95% CI: (36.42, 61.53). The analysis also indicated that there was substantial heterogeneity in included articles (I^2^ = 99.3%, p = 0.000) (Fig 2).

**Fig 3:**
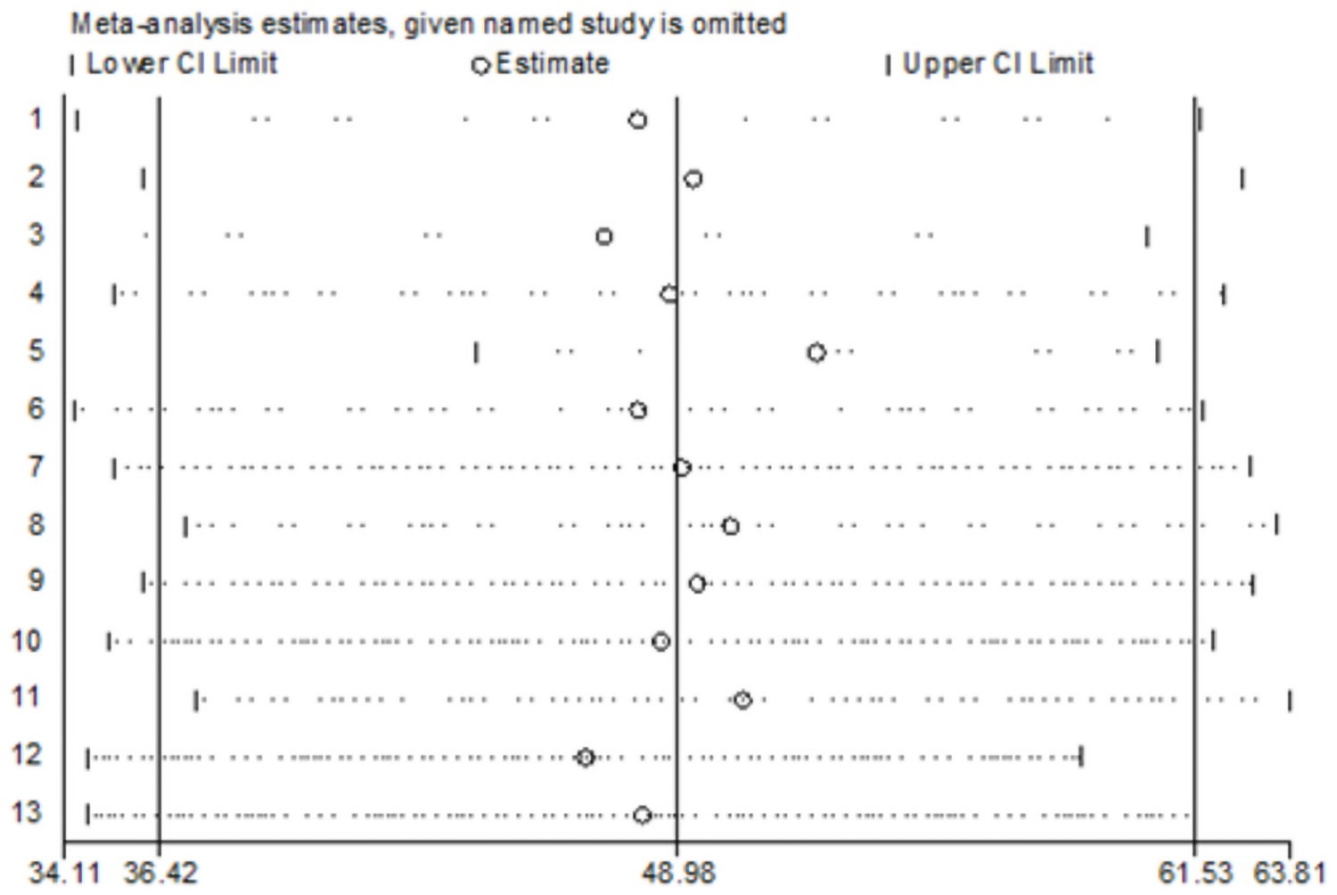
Sensitivity analysis for poor menstrual hygiene practice, a systematic review and meta-analysis, Ethiopia, 2020.

According to sensitivity analysis, there was no single influential estimate that significantly accounted for the observed heterogeneity (Fig 3).

**Fig 4:**
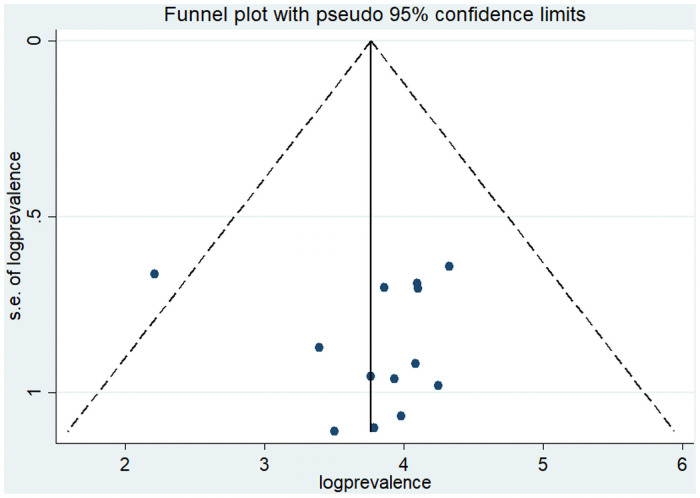
Funnel plot showing publication bias of prevalence of poor menstrual hygiene practice, a systematic review and meta-analysis, Ethiopia, 2020.

The funnel plot illustrated that there was a symmetrical distribution of studies (Fig 4).

Moreover, Egger’s test statistics indicated that there was no statistical evidence of publication bias (p = 0.702).

### Subgroup analysis

#### By the geographical regions and study setting

**Fig 5:**
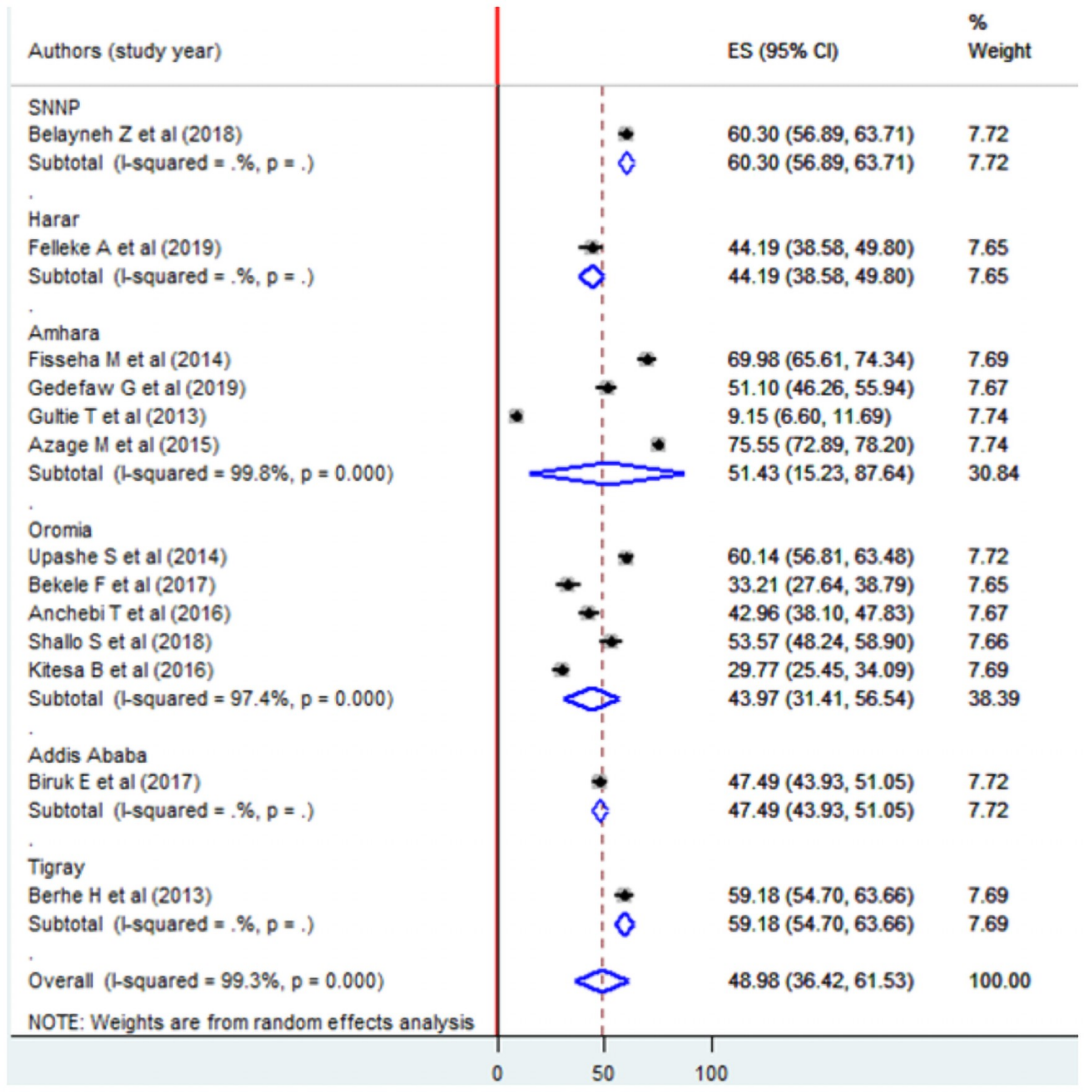
Forest plot of subgroup analysis by geographical zone (region) using the random effect model, a systematic review and meta-analysis, Ethiopia, 2020.

Subgroup analysis by a region where the studies were conducted indicated that the highest prevalence was observed in SNNPs, Ethiopia 60.3% (95% CI: 56.89, 63.71) and the lowest was in Oromia 43.97% (95% CI: 31.41, 56.54) (Fig 5).

**Fig 6:**
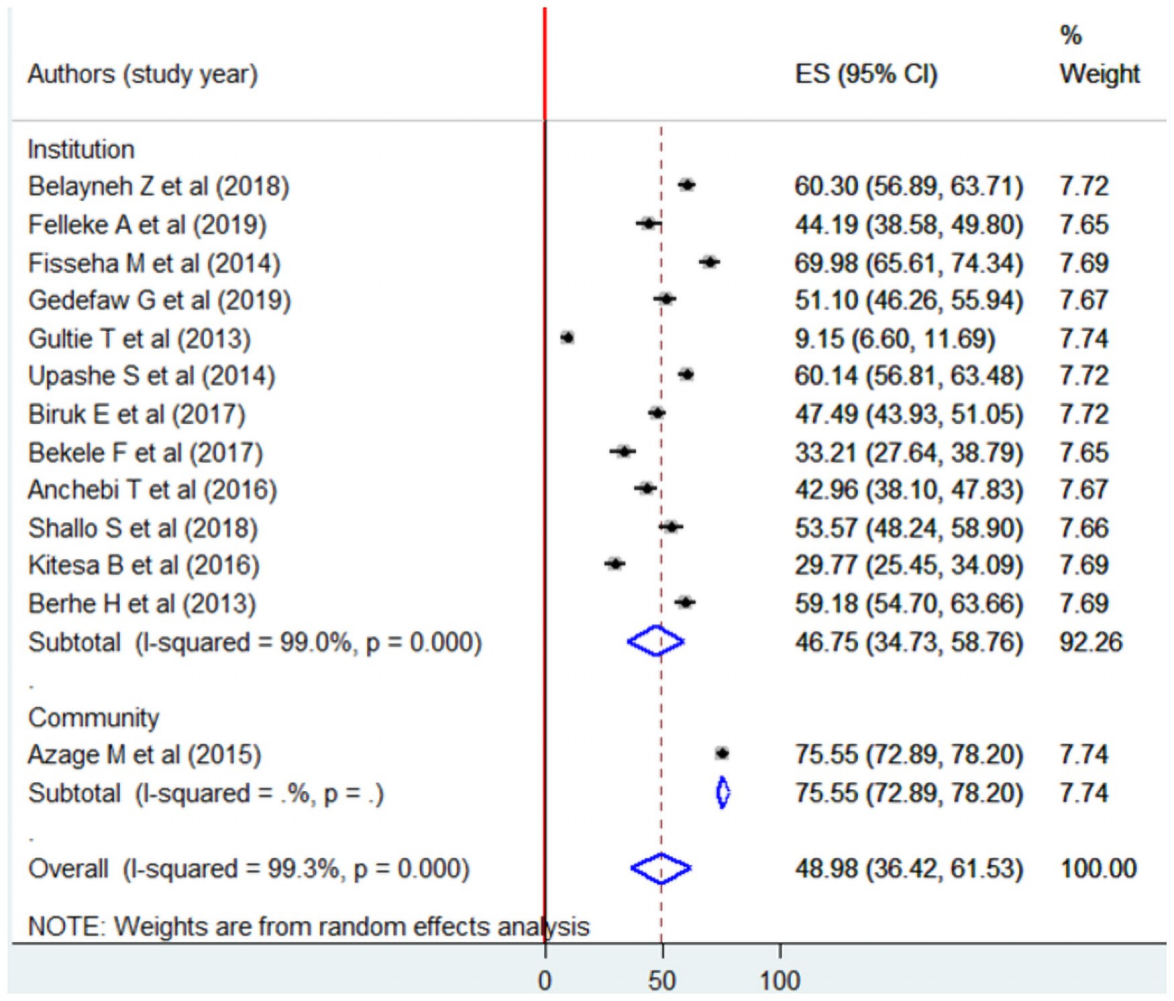
Forest plot of subgroup analysis by study setting using the random effect model, a systematic review and meta-analysis, Ethiopia, 2020.

Similarly, subgroup analysis by the study setting indicated that there was a statistical difference in menstrual hygiene practice among school and community-based studies [46.75%, 95% CI: (34.73, 58.76) and 75.55%, 95% CI: (72.89, 78.20) respectively] (Fig 6).

### The effect of knowledge on menstrual hygiene practice

**Fig 7:**
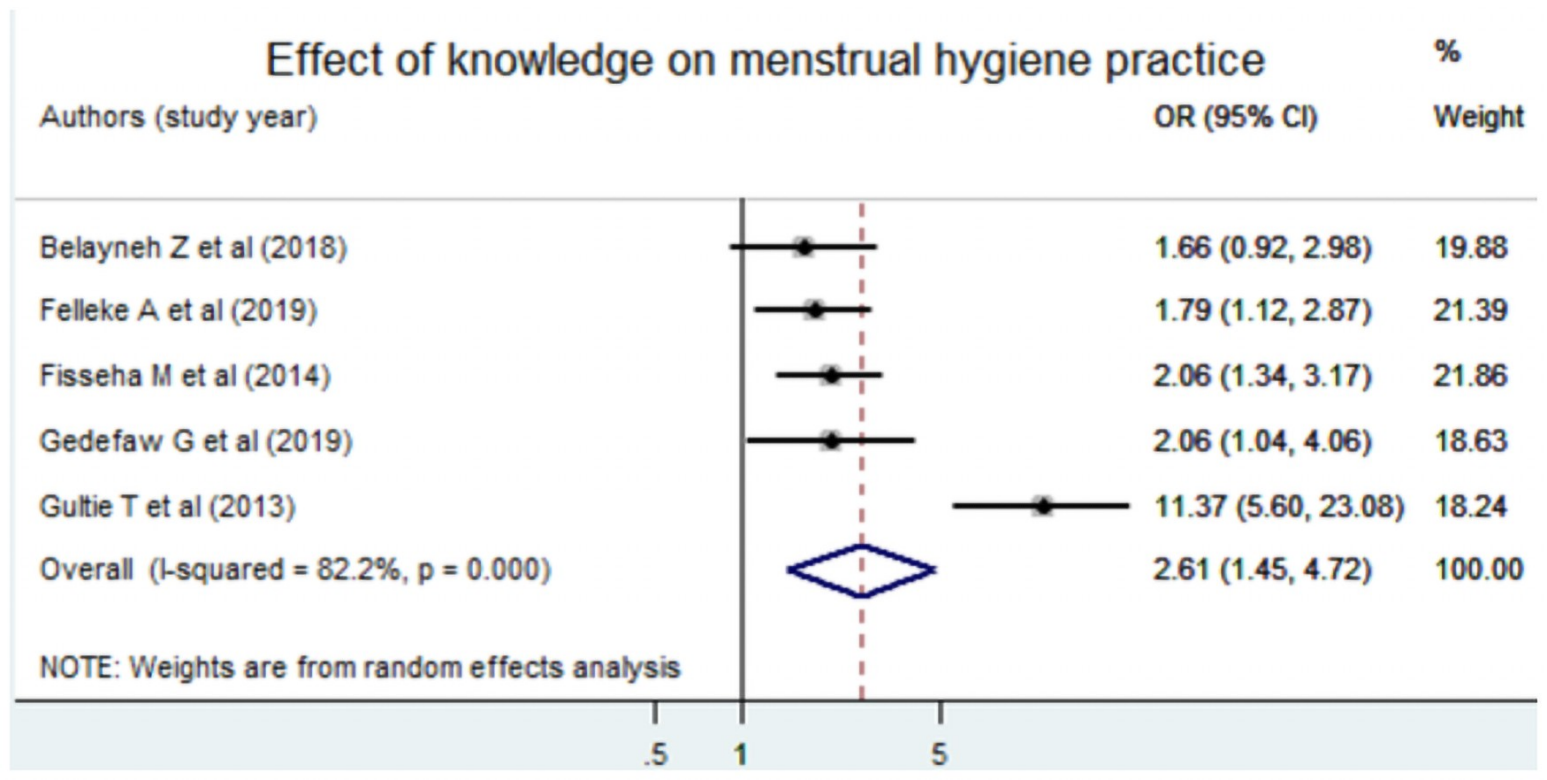
Forest plot of the pooled estimate of the effect of knowledge menstrual hygiene practice, a systematic review and meta-analysis, Ethiopia, 2020.

The effect of adolescent’s knowledge on menstrual hygiene practice was estimated from five individual articles and a random effect model was used. The pooled odds of poor menstrual hygiene practice among female adolescents who had poor knowledge regarding menstrual hygiene was increased by 2.6 as compared to counterparts [AOR = 2.61, 95% CI: (1.45, 4.72)] (Fig 7).

The egger test showed that there was no statistical evidence of publication bias (P value = 0.39) in the effect of knowledge on menstrual hygiene.

## Discussion

In this study, the pooled prevalence of poor menstrual hygiene practice among female adolescents in Ethiopia was 48.98% [95% CI: (36.42, 61.53)] and knowledge regarding menstrual hygiene was significantly associated with menstrual hygiene practice. The pooled prevalence of poor menstrual hygiene practice was in line with a study conducted in Uganda (45.45%) [25]. It was also in line with different studies conducted in India (44.8–60%) [21–23]. The finding of this result was consistent with a study conducted in Nepal (40%) [18].

But, it was low as compared to a study conducted in Nigeria (74.7%) [26] and Ghana (69.9%) [28]. It is also lower as compared to a study conducted in Bangladesh (68.5%) [20]. The possible reason for this observed discrepancy may be due to differences in cultural practices. Moreover, it may be due to the difference in social relationships and social value that the country gave for women in preserving their health.

Whereas it was high as compared to a study conducted in Kenya (28.8%) [27]. The finding was also high as compared to a study conducted in Nepal (27.5%) [19]. This difference may be due to the previous studies were among the pastoralist community and the current study was among the non-pastoralist community. The analysis also indicated that there was a significant difference in the prevalence of poor menstrual hygiene practice across the region in which the highest prevalence was observed in SNNPs, Ethiopia 60.3% and the lowest was in Oromia 43.97%. The difference may be due to cultural variation within the country since Ethiopia is a diversified country.

The pooled odds of poor menstrual hygiene practice among female adolescents who had poor knowledge regarding menstrual hygiene were increased by 2.6 as compared to counterparts. The finding was congruent with a study conducted in Kenya [27] and Ghana [28]. It is also supported by a study conducted in Pakistan [57]. The findings of studies conducted in Nepal [19] and Bangladesh [20] also witnessed the same thing (as the adolescents had poor knowledge they would have a high probability of having poor menstrual hygiene practice). This may be due to the fact that knowledge plays a great role and it is a prerequisite for practice [58]. Moreover, knowledge is one basic determinant of human behavior in the current context or theory of behavioral change.

Even though health is considered as a basic human right of everybody and it was the primary intervention area of the government, a significant number of female adolescents had poor menstrual hygiene practice. In relation to this practice, they may face different reproductive tract infections and other complications [11–14]. So, the adolescent themselves as well as the health care providers in any service delivery points should aware as it would be due to poor knowledge related to menstrual hygiene practice. Furthermore, teachers, parents and health care professionals and other interested agencies should be collaborated so as to resolve it. Lastly, further prospective and experimental studies should be the suggestive area of work for future researchers.

Despite the analysis had strength, it has certain limitations: All included articles were cross-sectional which may affect the overall point estimate. In addition, heterogeneity was not completely resolved in the final random effect model/analysis.

## Conclusions

The prevalence of poor menstrual hygiene practice was high and knowledge regarding menstrual hygiene was significantly associated with poor menstrual hygiene practice. Information education communication and behavioral change communication at all levels of education should be the primary focus area of the government. The health sectors should be also involved in creating awareness and helping female adolescents to have good knowledge order to improve menstrual hygiene practice.

## Supporting information

S1 File PRISMA checklist used in this systematic review and meta-analysis. (DOC)

S2 File Full electronic search strategy for PubMed data base. (DOCX)

S3 File Joanna Brigg’s Institute (JBI) critical appraisal for quality assessment. (DOCX)

S4 File The datasets used/analyzed in this systematic review and meta-analysis. (DTA)

S5 File The questions used to measure menstrual hygiene practice and knowledge. (DOCX)
