# Supplementary material for: Menstrual hygiene practice among female adolescents and its association with knowledge in Ethiopia: A systematic review and meta-analysis
Source: PLoS One. 2021 Aug 4;16(8):e0254092. doi: 10.1371/journal.pone.0254092 (PMC8336879; doi:10.1371/journal.pone.0254092)
Supplement: S2 File — (DOCX) [file pone.0254092.s002.docx]

((((((Menstruation AND ((journalarticle[Filter]) AND (humans[Filter]) AND (female[Filter]) AND (english[Filter]) AND (adolescent[Filter]))) OR (menstrual hygiene AND ((journalarticle[Filter]) AND (humans[Filter]) AND (female[Filter]) AND (english[Filter]) AND (adolescent[Filter])))) OR (menstrual hygiene practice AND ((journalarticle[Filter]) AND (humans[Filter]) AND (female[Filter]) AND (english[Filter]) AND (adolescent[Filter])))) OR (knowledge on menstrual hygiene AND ((journalarticle[Filter]) AND (humans[Filter]) AND (female[Filter]) AND (english[Filter]) AND (adolescent[Filter]))) AND ((journalarticle[Filter]) AND (humans[Filter]) AND (female[Filter]) AND (english[Filter]) AND (adolescent[Filter]))) AND ((((factors AND ((journalarticle[Filter]) AND (humans[Filter]) AND (female[Filter]) AND (english[Filter]) AND (adolescent[Filter]))) OR (factors associated AND ((journalarticle[Filter]) AND (humans[Filter]) AND (female[Filter]) AND (english[Filter]) AND (adolescent[Filter])))) OR (risk factors AND ((journalarticle[Filter]) AND (humans[Filter]) AND (female[Filter]) AND (english[Filter]) AND (adolescent[Filter])))) OR (predictors AND ((journalarticle[Filter]) AND (humans[Filter]) AND (female[Filter]) AND (english[Filter]) AND (adolescent[Filter]))) AND ((journalarticle[Filter]) AND (humans[Filter]) AND (female[Filter]) AND (english[Filter]) AND (adolescent[Filter])))) AND ((((females AND ((journalarticle[Filter]) AND (humans[Filter]) AND (female[Filter]) AND (english[Filter]) AND (adolescent[Filter]))) OR (school girls AND ((journalarticle[Filter]) AND (humans[Filter]) AND (female[Filter]) AND (english[Filter]) AND (adolescent[Filter])))) OR (women AND ((journalarticle[Filter]) AND (humans[Filter]) AND (female[Filter]) AND (english[Filter]) AND (adolescent[Filter])))) OR (adolescents AND ((journalarticle[Filter]) AND (humans[Filter]) AND (female[Filter]) AND (english[Filter]) AND (adolescent[Filter]))) AND ((journalarticle[Filter]) AND (humans[Filter]) AND (female[Filter]) AND (english[Filter]) AND (adolescent[Filter]))) AND ((journalarticle[Filter]) AND (humans[Filter]) AND (female[Filter]) AND (english[Filter]) AND (adolescent[Filter]))) AND (Ethiopia AND ((journalarticle[Filter]) AND (humans[Filter]) AND (female[Filter]) AND (english[Filter]) AND (adolescent[Filter])))
