# Supplementary material for: Menstrual hygiene practice among female adolescents and its association with knowledge in Ethiopia: A systematic review and meta-analysis
Source: PLoS One. 2021 Aug 4;16(8):e0254092. doi: 10.1371/journal.pone.0254092 (PMC8336879; doi:10.1371/journal.pone.0254092)
Supplement: S3 File — (DOCX) [file pone.0254092.s003.docx]

| Descriptive cross sectional studies | | Articles | | | | | | | | | | | | |
| --- | --- | --- | --- | --- | --- | --- | --- | --- | --- | --- | --- | --- | --- | --- |
| JBI Checklists | | Belayneh Z et al | Felleke A et al | Fisseha M et al | Gedefaw G et al | Gultie T et al | Upashe S et al | Biruk E et al | Bekele F et al | Anchebi T et al | Shallo S et al | Kitesa B et al | Azage M et al | Berhe H et al |
| 1 | Was the sample frame appropriate to address the target population? | 1 | 0 | 0.5 | 1 | 0 | 0.5 | 0 | 0 | 0 | 0 | 0 | 0.5 | 0 |
| 2 | Were study participants sampled in an appropriate way? | 1 | 0.5 | 0.5 | 0.5 | 0.5 | 1 | 1 | 0.5 | 1 | 0.5 | 0.5 | 1 | 0 |
| 3 | Was the sample size adequate? | 1 | 0.5 | 1 | 1 | 0.5 | 1 | 1 | 0.5 | 1 | 0.5 | 0.5 | 1 | 1 |
| 4 | Were the study subjects and the setting described in detail? | 0.5 | 0.5 | 0.5 | 1 | 0.5 | 0.5 | 0.5 | 1 | 0.5 | 0.5 | 0.5 | 1 | 0.5 |
| 5 | Was the data analysis conducted with sufficient coverage of the identified sample? | 1 | 1 | 0.5 | 1 | 1 | 1 | 1 | 0.5 | 1 | 1 | 1 | 1 | 1 |
| 6 | Were valid methods used for the identification of the condition? | 1 | 0.5 | 0.5 | 0 | 0.5 | 1 | 1 | 1 | 1 | 0.5 | 0.5 | 0.5 | 0.5 |
| 7 | Was the condition measured in a standard, reliable way for all participants? | 0.5 | 0 | 0.5 | 0.5 | 1 | 1 | 0.5 | 0.5 | 0.5 | 0.5 | 1 | 1 | 1 |
| 8 | Was there appropriate statistical analysis? | 1 | 1 | 1 | 1 | 1 | 1 | 1 | 1 | 1 | 1 | 1 | 1 | 1 |
| 9 | Was the response rate adequate, and if not, was the low response rate managed appropriately? | 1 | 1 | 1 | 1 | 1 | 1 | 1 | 1 | 1 | 0.5 | 1 | 1 | 1 |
| Total JBI score | | 8 | 5 | 6 | 7 | 6 | 8 | 7 | 6 | 7 | 5 | 6 | 8 | 6 |
