# Supplementary material for: Menstrual hygiene practice among female adolescents and its association with knowledge in Ethiopia: A systematic review and meta-analysis
Source: PLoS One. 2021 Aug 4;16(8):e0254092. doi: 10.1371/journal.pone.0254092 (PMC8336879; doi:10.1371/journal.pone.0254092)
Supplement: S5 File — (DOCX) [file pone.0254092.s005.docx]

| S.NO | Question to measure menstrual practice | Response | Remark |
| --- | --- | --- | --- |
| 1 | Using absorbent materials during menstruation | 1. No 2. Yes |  |
| 2 | Using commercially made sanitary pad during menstruation | 1. No 2. Yes |  |
| 3 | Change pads or cloths more than three times a day during menstruation | 1. No 2. Yes |  |
| 4 | Using clean clothes with soap and water | 1. No 2. Yes |  |
| 5 | Dries cloths in sunlight | 1. No 2. Yes |  |
| 6 | Cleans external genitalia during menstruation | 1. No 2. Yes |  |
| 7 | Disposes the pads by wrapping with paper | 1. No 2. Yes |  |
| 8 | Washes bath daily with soap during menstruation | 1. No 2. Yes |  |
| 9 | Cleaning of external genitalia with water and soap during menstruation | 1. No 2. Yes |  |
| 10 | Disposes used sanitary pads in dustbin | 1. No 2. Yes |  |

| S.NO | Question to measure knowledge related to menstrual practice | Response | Remark |
| --- | --- | --- | --- |
| 1 | It is a lifelong process | 1. No 2. Yes |  |
| 2 | It is pathological | 1. No 2. Yes |  |
| 3 | It has foul smell | 1. No 2. Yes |  |
| 4 | It is a sign of conception | 1. No 2. Yes |  |
| 5 | The causes of menses bleeding is hormonal change | 1. No 2. Yes |  |
| 6 | It is a normal phenomenon | 1. No 2. Yes |  |
| 7 | It is unique to females | 1. No 2. Yes |  |
| 8 | Its sources of bleeding is uterus | 1. No 2. Yes |  |
